# Challenges and motivating factors for integrating geostatistical models in targeted schistosomiasis control: A qualitative case study in Northwestern Tanzania

**DOI:** 10.1371/journal.pntd.0012770

**Published:** 2024-12-30

**Authors:** Jake D. Mathewson, Linda van der Spek, Dunstan J. Matungwa, Anna Samson, Harry L. S. Coleman, Ente J. J. Rood

**Affiliations:** 1 KIT Royal Tropical Institute, Epidemiology, Center for Applied Spatial Epidemiology (CASE), Amsterdam, The Netherlands; 2 National Institute for Medical Research (NIMR), Mwanza, Tanzania; 3 School of Medicine, Department of Medical Parasitology & Entomology, Catholic University of Health and Allied Sciences, Mwanza, Tanzania; 4 KIT Royal Tropical Institute, Health Systems Strengthening, Amsterdam, The Netherlands; George Washington University School of Medicine and Health Sciences, UNITED STATES OF AMERICA

## Abstract

**Introduction:**

To address problems of over- and under-treatment with preventive chemotherapy resulting in ongoing transmission of schistosomiasis, the World Health Organization (WHO) recommends targeted mass drug administration (MDA) interventions at a sub-district level. In Tanzania, the lack of sub-district (ward) prevalence data has inhibited a transition to targeted treatment. Model-based prevalence estimation combined with routine surveillance data can be used to overcome this gap. We created a geostatistical model to estimate parasitological prevalence in the wards of the Lake Zone regions of Tanzania to investigate opportunities for enabling targeted MDA for schistosomiasis. With no precedent on how outputs from a geostatistical model could be used to inform decision-making in Tanzania, this qualitative study explores perceptions on what may challenge and motivate program staff in Tanzania’s national schistosomiasis control program to integrate the models into routine planning to guide disease control interventions.

**Methods:**

Seven semi-structured, key informant interviews were conducted in 2022 examining perceived programmatic challenges and motivations of integrating the geostatistical model into current programming through various thematic areas: information systems, financing, services and operational capacity, policy and planning, and coordination. Key informants included decision-making staff in the Ministry of Health’s neglected tropical diseases (NTD) control program, WHO NTD staff, schistosomiasis MDA implementing partners, academic experts studying the control of schistosomiasis, and central-level NTD coordinators.

**Results:**

Informants unanimously acknowledged that the geostatistical model could be useful in guiding targeted interventions, and found several factors that may motivate programmatic uptake including providing a financially feasible method to comprehensive prevalence estimates, facilitation of essential implementation tasks like site selection for MDA and screening, as well as annual calculation of treatments required for requesting medicine. Key challenges to integration were seen in limitation of existing modeling expertise, sensitization, and most importantly in the lack of WHO recommendations surrounding model use, as national disease control strategies and policies are built around WHO guidelines.

**Conclusions:**

Geostatistical models like the one presented can feasibly be integrated in decision-making for targeted interventions based on domestic capacity, financial availability and readiness. However, the lack of WHO guidance on the use of these tools calls for action to translate the potential of such models into recommendations that encourage routine integration from national programs. Overcoming this key inhibiting factor will be a crucial first step toward the integration of such models.

## Author summary

Schistosomiasis remains an extremely prevalent disease and a major public health problem in Tanzania despite years of disease control interventions like mass drug administration (MDA). While these interventions have expanded in scale and geographic coverage over the past 15 years, they still fail to reach millions of people who are estimated to need treatment with preventive chemotherapy (PC) drugs, resulting in severe morbidities and death secondary to schistosomiasis infection, and continued disease transmission. In its treatment guidelines, the World Health Organization (WHO) has advocated for national programs to move to a more targeted method of treatment to enable more effective and efficient use of limited resources. Problematically, decision makers in Tanzania don’t have disease prevalence data for much of the country that would allow them to plan for such targeted distribution of PC in Tanzania.

In 2024, KIT Royal Tropical Institute published a study on how disease prevalence estimates from geostatistical models can be used to fill these inhibitive data gaps and allow country programs to move to targeted treatment as advocated for in the WHO guidelines. With no clear precedent on how use of such models would be integrated into existing schistosomiasis control programs, this study sought to examine perceptions of challenges and incentives to programs associated with integrating these models into the planning process in Tanzania. Through interviews with key informants involved in the planning of schistosomiasis control in Tanzania, the study finds clear actionable barriers to integrating such models in existing systems, despite their perceived utility to support the planning of targeted interventions.

In this study, we explore challenges and motivating factors of using this model to better understand blockers inhibiting routine use of such models across highly endemic countries like Tanzania that are stagnating on elimination targets and unable to move to targeted interventions. By examining motivating factors, we further seek to understand what encourages programmatic uptake of models to facilitate their eventual integration into health systems in endemic areas.

We believe that results from this study should help to motivate the WHO to standardize recommendations around using geostatistical models, like the one presented in this study, which may serve as powerful tools to better plan interventions and reduce transmission of schistosomiasis. Such recommendations may, as this study found, have a large influence on country uptake of novel tools at country level, helping them to combat a devastating disease that affects so many millions of people across Sub-Saharan Africa and other parts of the world.

## Background

Schistosomiasis is a debilitating neglected tropical disease (NTD) caused by parasitic blood flukes that enter the body following contact with contaminated fresh water [1]. Estimates on annual global deaths due to schistosomiasis vary widely between 12,000 and 200,000 people annually, with over 700,000 people believed to be at risk of infection and manifesting numerous morbidities associated with the disease [2,3]. Schistosomiasis is a severe public health problem in Tanzania; the country with the second highest burden in the world [4]. In spite of ongoing control efforts to reduce transmission, upwards of 50% of the country’s population are either exposed or living within an area with a high risk of exposure [4]. Due to the large number of people and communities that schistosomiasis continues to affect, the World Health Organization (WHO) has prioritized the elimination of schistosomiasis as a public health problem by the year 2030 [5].

In the past decade, the primary control measure for reducing schistosomiasis transmission has been through mass drug administration (MDA) with the preventive chemotherapy (PC) drug praziquantel in districts determined to be endemic [3,6]. Treating whole districts, however, has proven to be a resource-intensive practice that has led to the unnecessary treatment of populations with little or no disease exposure (over-treatment), while also failing to treat many infected individuals and communities living in districts where either prevalence has never been surveyed or were deemed to be “non-endemic” through school sampling (under-treatment) [7]. As schistosomiasis has been demonstrated to be distributed heterogeneously throughout districts, clustering in areas with certain environmental and socio-demographic characteristics, the WHO has recommended that country programs conduct more targeted MDA treatment campaigns at a sub-district level to reduce issues of over- and under-treatment [5,6].

In addition to recommendations on conducting more targeted treatment, the WHO also provides explicit guidelines for how frequently to treat communities based on disease prevalence and intensity of infection [5]. The practice of planning targeted interventions, however, is extremely challenging for countries to adhere to with scarcely available parasite prevalence data at sufficient spatial resolutions required to make decisions regarding targeted PC treatment. Therefore, the granular mapping of schistosomiasis transmission focal points is considered a prerequisite to enable the planning of higher impact and more efficient interventions [8]. This mapping, often termed precision mapping, seeks to more comprehensively screen high-risk groups than in conventional district level sampling to highlight areas of high transmission within districts, and to enable more targeted treatment and avoid the aforementioned pitfalls of district level aggregation and treatment [8]. Precision mapping may entail different methodologies in different contexts. A 2021 precision mapping study was conducted in north-western Tanzania, the first parasitological prevalence testing in the region since 2004/2005, which evaluated 10 wards or clusters within 29 different districts in ecological zones deemed to be high risk for schistosomiasis transmission [9]. Access to such granular disease prevalence data can be very beneficial for planning interventions for schistosomiasis control. Although the presence of schistosomiasis infection has been well documented around the Lake Zone bordering Lake Victoria in north-western Tanzania, there is limited data on the distribution of the disease at lower administrative levels within districts, as well as throughout many other parts of the country [10–12].

A major limitation with transitioning to a precision mapping strategy is that in spite of its improved capacity to identify focal areas of schistosomiasis transmission, routine and comprehensive precision mapping is likely too costly for country programs to sustain [8,9]. In *A road map for neglected tropical diseases 2021–2030*, the WHO acknowledges that attempting comprehensive precision mapping on a recurring basis throughout endemic countries would not be financially feasible, and that “more cost effective mapping strategies are necessary for targeting preventive chemotherapy” [13]. Without suggesting alternative methods, the guide simply states that “new approaches and mapping tools are necessary to obtain a granular view of disease epidemiology and progression for targeted intervention” [13].

Geostatistical models have been examined in this context, and have demonstrated to be a viable and cost effective method of bridging data scarcity gaps with model estimates [14]. In spite of these findings and other studies demonstrating the utility of the use of geostatistical models for estimating schistosomiasis prevalence [15–17], country programs have largely not implemented a geostatistical approach, or a variety of other modeling approaches for that matter, to estimate prevalence at smaller spatial scales allowing them to transition to targeted disease control interventions. Currently there is a paucity of research seeking to understand the existing barriers that inhibit schistosomiasis control professionals from integrating geospatial tools, such as geostatistical models, into routine programming. Without this information, modeling groups, largely based in the global north, continue to demonstrate the uses of geostatistical models with limited understanding of factors encouraging and inhibiting their uptake by national control programs in endemic settings. A better understanding of the challenges inhibiting national programs from adopting these models is urgently needed to facilitate uptake of various models that can be used to support the eventual transition to sub-district, targeted treatment.

Together with a consortium of Tanzanian and European-based partners, the Center for Applied Spatial Epidemiology at KIT Royal Tropical Institute created a geostatistical model, using the outputs of the precision mapping done in 2021, to predict parasitological prevalence in wards of the Lake Zone regions of Tanzania to examine their eligibility for treatment under existing WHO guidelines [7]. In Tanzania, wards are two levels of administration below districts. The resulting study estimated ward-level prevalence throughout the Lake Zone districts to facilitate targeted decision-making for MDA in spite of existing data scarcity issues. While other spatial and geostatistical models for estimating schistosomiasis prevalence have been developed [14,16,18–23], few NTD programs in high burden countries have integrated such tools into their decision making processes for MDA planning.

To understand why geostatistical models are not being used to guide targeted schistosomiasis interventions in Tanzania, this study explores programmatic perceptions of integrating such a model into routine planning. Through key informant interviews (KII), the study specifically investigates: 1) perceived challenges associated with the novel geostatistical model’s use, 2) factors that may motivate its integration, and 3) whether the model could facilitate a transition to targeted intervention planning. The study acknowledges that integrating the geostatistical model into Tanzania’s schistosomiasis and MDA planning processes, as well as the broader health information system, would be a complex change process. Consequently, it examines these research questions from various functional perspectives to capture the full range of perceptions. While focused on a specific geostatistical model in Tanzania, the study aims to contribute to the broader understanding of why such models are not being adopted in schistosomiasis-endemic countries across Africa.

## Methods

### Ethics statement

In this study, all participants provided both written and verbal consent prior to each interview. This was done first through a consent form which was emailed back to the researchers before the interview, and verbally once the recording had started at the onset of the interview. The study team assured the participants that the data from this study would be confidentially handled and wherever and whenever the data from this study would be used; their identities would be anonymized by using code numbers. All informants received the additional opportunity to verify quotes and ultimately provide a final consent prior to submission of the manuscript.

Given that the interviews were conducted among working professionals and therefore determined to be low risk, the Research Ethics Committee at the Royal Tropical Institute (KIT) granted the study a waiver (S-228) from full ethical review. Permission for the use of secondary data to demonstrate quantitative models and mapping data during interviews was granted by Tanzania’s Ministry of Health (Ref.No. MB.87/266/01/128).

### Study approach and theory

**Fig 1:**
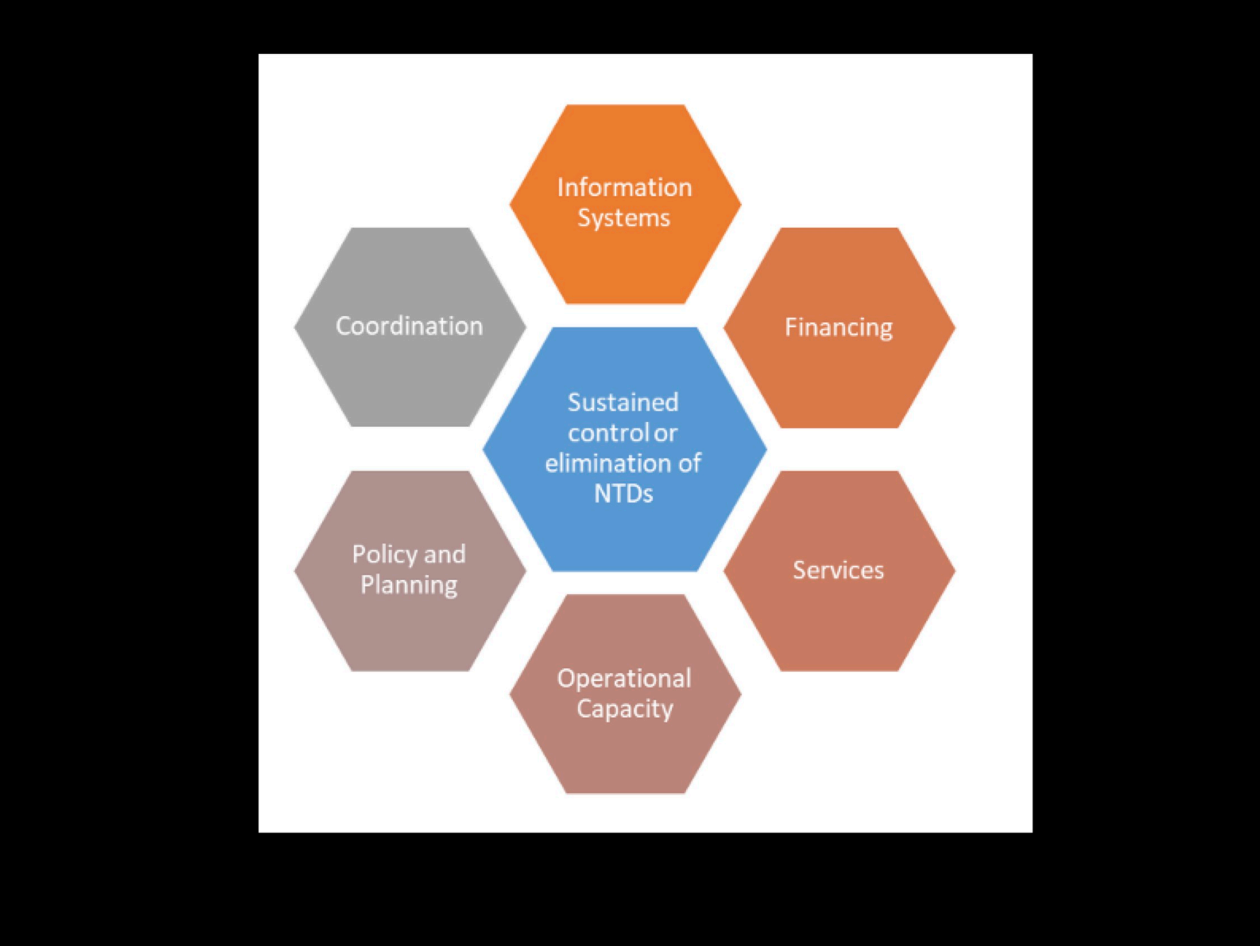
Functional areas for sustained control or elimination of NTDs, readapted from the USAID NTD Sustainability Framework **Plan** for the control and elimination of NTDs [26].

A qualitative case study approach [24] was used to investigate the motivating factors and challenges associated with integrating the proposed model to support targeted planning of MDAs in Tanzania. Two frameworks informed the theory of the study: the Dalberg sustainability assessment tool and the USAID NTD sustainability framework. The Dalberg sustainability assessment tool [25] was initially used in developing a topic sheet to guide interviews with the participants. The framework sees NTD program sustainability as government ownership across six dimensions of sustainability: policy and leadership, budget, the delivery system, organizational capacity, partnerships, and evaluation and adaptation. The USAID NTD sustainability framework (Fig 1) was later used to categorize themes discussed with informants across its “functional areas”: financing, services, information systems, operational capacity, policy and planning, and coordination [26]. These functional areas are components of NTD programming that, together, lead to sustained NTD control and elimination. The framework implies that each of these functional areas requires government ownership and integration in existing systems in order to achieve sustained control and elimination. For the purposes of this study, the functional areas [26] classify the relevant issues articulated by interviewed participants on: the perceived challenges and motivating factors associated with the integration of the proposed geostatistical model in current NTD decision-making processes, and whether participants believed that the model can support a transition to sub-district targeted intervention planning. These functional areas are highlighted in Table 1, with a description of how they appear in the USAID framework and how they were used in the context of this study.

**Table 1:** Description of the distinct USAID functional areas that lead to sustained control or elimination of NTD, and how the different themes applied to this study.

Functional area	Definition per USAID NTD framework	Use in this study
**Coordination**	Coordination of NTD interventions across relevant health (malaria, maternal and child health) non-health (WASH, education) sectors, with a view to mainstreaming NTD programs in existing delivery platforms	Coordination of drugs distributed in schistosomiasis MDA with other PC-NTDs, like soil transmitted helminths (STH).
**Policy and planning**	Integration of core NTD functions in national health policies and sector strategies, and considering the link between NTD control and elimination and poverty alleviation, gender and social context	Assessing the capacity of national strategic plans and documents for MDA programming for schistosomiasis, as well as WHO recommended guidelines for the distribution of preventive chemotherapy.
**Operational capacity**	Functioning and optimized procedures—such as drug donation, procurement, distribution, diagnostics—that uphold NTD programs, including the availability of inputs (human resources, medicines, supplies), with government oversight and integration in health system supply chains to the extent possible. Also includes the ability to conduct surveillance and disease assessments, and forecast needs.	Understanding ability of planning and field teams to use geostatistical model outputs to provide actionable recommendations for targeted MDA, as well as better informed targeted of future precision mapping.
**Information systems**	Integration of NTD indicators in national and routine health information systems, with reporting and use at facility and local government levels	Capacity of the existing system to generate data that can be integrated into future models, as well as to integrate model outputs in the context of existing data to help guide interventions.
**Services**	Provision of NTD interventions through existing and new community and facility platforms, through its inclusion in minimum service packages and targeting for groups traditionally missed by MDA	Combined with operational capacity section.
**Financing**	Mobilization of domestic funding for and allocation of government budgets towards NTD interventions, mainstreamed into budgeting and planning mechanisms at national and local levels	Investigating the perceived implications of integrating the geostatistical models on the existing budget for the national schistosomiasis control program in Tanzania.

### Sampling and recruitment

**Fig 2:**
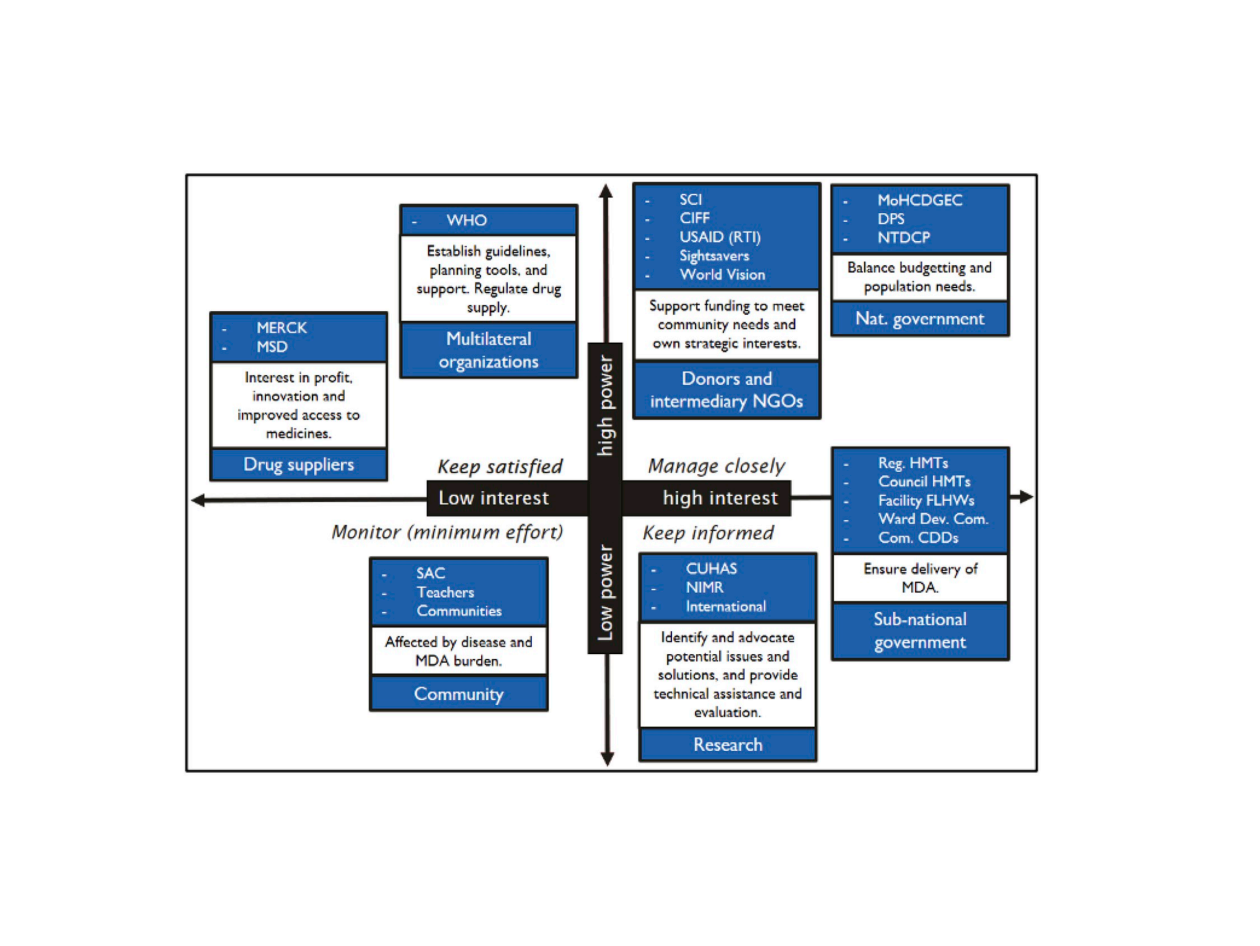
Primary stakeholder groups involved in and affected by schistosomiasis control activities, along with their interest drivers. A clockwise description of the stakeholders present: MERCK pharmaceuticals and its international subsidiary MSD are shown under drug suppliers, the World Health Organization (WHO) is represented under multilateral organizations. Donors and intermediary NGO’s include Schistosomiasis Control Initiative (SCI, now ’Unlimit Health’), Children’s Investment Fund Foundation (CIFF), and USAID (through a RTI International led program). Under the national government stakeholders in the top right corner, the Ministry of Health (MoH), development partners for the ministry (DPS), and the national neglected tropical disease control program (NTDCP). Subnational government stakeholders include regional and council level health management teams (HMTs), facility level health workers (FLHWs), Ward Development Committees (Ward Dev. Com.), and community drug distributors (CDDs). Research organizations include the Catholic University for Health and Allied Sciences (CUHAS) and Tanzania’s National Institute for Medical Research (NIMR). Among members of the community, SAC stands for school aged children, who have historically been viewed as the most high-transmission age group for schistosomiasis.

To inform sampling and recruitment of the study participants, we first mapped and conducted preliminary discussions with some stakeholders involved in the MDA decision-making in Tanzania. The mapping exercise revealed that there are relatively few planners involved in MDA planning and decision-making in mainland Tanzania. All stakeholders were mapped by decision making power and interest in the selection of MDA locations. Fig 2 shows the preliminary mapping of stakeholders prior to the interviews, which subsequently helped to inform the recruitment of participants.

Following the mapping exercise, purposive sampling was used to select key informants with varying levels of involvement in MDA programming and implementation for schistosomiasis control in Tanzania. Informants were selected based upon proximity to planning and monitoring of MDA, with participants recruited from different levels of the MDA planning process. Participants included staff of the national schistosomiasis control program of Tanzania, academic experts, local implementing partners, and representatives from the WHO. The number of study participants is limited to seven because there are a few planners involved in the decision-making process for schistosomiasis MDA selection in Mainland Tanzania, a fact that became clear during the stakeholder mapping exercise. The sample of seven key informants was additionally determined to be sufficient to attain saturation based on previous research that had demonstrated that four to six key informant interviews are not only enough to attain saturation, but in some cases generate as much relevant data as 12–15 interviews [27]. Table 2 provides an overview of the key informants’ roles regarding schistosomiasis control in Tanzania.

**Table 2:** Roles of Key Informants.

Role	Code	n
Academic Experts	AE1, AE2	2
Tanzania-based Implementing Partners	IP1, IP2	2
Tanzania Neglected Tropical Disease Country Program (NTDCP) staff	NTDCP1, NTDCP2	2
WHO NTD staff	WHO	1
**Total**		**7**

### Data collection

Data collection involved semi-structured interviews conducted with selected key informants (annex 1). At the beginning of the interviews, participants were first asked about national capacity to conduct sub-district level interventions with current tools available. Following these, participants were given a standardized introduction to the topic and a short presentation on the development of the model and its outputs. The generic semi-structured questionnaire is shown in S1 Text, which was slightly adapted for each interview depending on the role of the participant. The geostatistical model was presented to participants as part of the interview. In the model, ward-level prevalence estimates were generated through combining a zero-inflated Poisson model and kriging approach (regression kriging). To make predictions, the model used prevalence survey data collected in 2021 of more than 17,000 school children in six regions of Tanzania, along with several open source ecological and socio-demographic variables with known associations with schistosomiasis [7]. Further details of how the model was structured along with its outputs are published in a separate PLOS NTD manuscript [7].

Key informants were: 1) given a demonstration of the data sources and covariates that went into the model, and how each one associated with disease prevalence per the 2021 precision mapping study (S1 Fig), 2) provided with a map of wards where the model estimated mean prevalence to exceed the 10% treatment cutoff in the Lake Zone of Tanzania (S2 Fig), and 3) shown estimates of the implications for those ward level estimated treatment needs expressed in terms of number of treatments required, and how these estimates differed from conventional district level treatment needs (S3 Fig). Finally, informants were asked questions relating to the perceived challenges and motivating factors for the models implementation through the lens of different topics of the framework [28]. Interviews were given by one of two researchers (JM and LvdS) who closely coordinated using the topic guide and notes from previous interviews. All interviews were conducted in English and online using Microsoft Teams, where they were audio recorded and transcribed through the application’s live transcription feature. The duration of the interviews ranged between 60 and 100 minutes. To ensure fidelity and accuracy of the information that had been transcribed, researchers reviewed and extensively edited all the transcripts using playbacks of audio recording.

### Data analysis

Transcripts were coded using a combination of inductive and deductive approaches. Initially, interview transcripts were deductively coded among the corresponding functional areas of the USAID NTD sustainability framework. This was followed by inductive coding where transcripts were further reviewed to identify other themes and patterns in the data, particularly paying attention to opportunities and challenges in integrated model use that may have fit outside of the categories of the framework. The final codes were again reviewed and analyzed to articulate the barriers and enablers to integrating new tools in MDA decision-making and planning processes.

## Results

The perceptions of key informants are organized by the themes of the USAID NTD sustainability framework. They seek to organize responses by informants into the various thematic areas to explore the different dimensions of factors that can serve to motivate or challenge programs to integrate the geostatistical models to guide targeted schistosomiasis control interventions in Tanzania. Table 2 provides a summary of key findings from the interviews.

### Information systems

There was a unanimous agreement among all informants that the scarcity of schistosomiasis prevalence data was a key factor that has, up until now, inhibited the NTD Country Program (NTDCP) from transitioning to targeted ward-level MDA.

A number of informants described how the absence of prevalence data has resulted in treating some districts, with no empirical evidence that the district exceeds the prevalence threshold required for PC treatment:

“We don’t have any target, just we are giving drugs to anyone, we don’t know which communities [are] at high risk, which communities [are] at low risk…If we could have countrywide mapping, and then we identify communities which need different strategies for the MDA, that will be very help[ful]. But that is not there in the in the country…How do you have a focused MDA when you don’t know where the infection is occurring?”–AE1

Informants felt that in the absence of countrywide precision mapping, access to the geostatistical model outputs could allow for more data informed targeting than currently being employed. Informants discussed how limited access to data currently manifests in making large assumptions about the disease prevalence across geographic areas in Tanzania:

“…in many cases even there wasn’t survey data available at implementation level, so the countries basically extrapolate the information from neighboring implementation units to [other] implementation units that were not mapp[ed] yet. They simply…say OK, … [in] these areas the endemicity is high, so we assume that in those…other neighboring areas that the endemicity is high too.”–WHO

“We have … quite a number of communities which have no data but [are] assigned the aggregated endemicity, from the district level. …It’s actually…often not the right…endemicity.”–NTDCP1

Such examples from interviewed participants highlight the challenge that lacking disease prevalence data poses to switching to more targeted planning of MDA. Prevalence data is not only lacking on a ward level in Tanzania, but also often on a district level, which has necessitated alternative methods for MDA site selection. Informants acknowledged that this creates incentives for using prediction models like the geostatistical model demonstrated in the interview:

“…[a] prediction model can help get the right endemicity for those communities and therefore [help to] plan accordingly.”–NTDCP1

“I see the benefit… It helps those who plan the MDA to see which ward needs some intervention [and] which one does not.”–NTDCP2

When reflecting on the sources of information and specific covariates that the geostatistical model uses to generate predictions, some informants cautioned that ecological data sources can be prone to error, as they may be change over time. Informants also acknowledged challenges in access to reliable demographic data, which can furthermore introduce error in the model’s predictions. While informants posited that such limitations could reduce confidence in the models estimates, no informants cited them as a rationale for not integrating the model to support current efforts to better target interventions.

### Financing

The informants furthermore explored the financial implications associated with using the model. Several informants acknowledged that a key reason for the paucity of prevalence data at the ward level in Tanzania was the expense of conducting routine precision mapping surveys, normally conducted in schools across districts, in a program with limited financial resources. These informants acknowledged that although there is a need for ward-level prevalence data, conducting comprehensive precision mapping to get them would not be financially feasible.

“Without modeling, that means you are carrying…the entire cost. You’re [testing] every school…that means you need to reach…every school in the country.”–AE1

“We need to start thinking [about] the most cost-effective approach for this precision mapping [to] balance the cost of conducting many surveys.”–WHO

As reaching each school in the country on an annual basis would be a challenge from both financial and operational perspectives, the informants acknowledged a need for creating alternative strategies to estimate prevalence. This provides further incentive for programs to supplement their limited existing data with model predictions. Informants largely agreed that the use of the geostatistical model to supplement precision mapping estimates would be a more cost-effective approach to having a more comprehensive understanding of prevalence in all wards across the country. When discussing with an academic expert, they explained that the model outputs shown could be:

“… very helpful in terms of drug distribution, in terms of costing, it can reduce a lot of cost as we plan for the targeted… MDA.”–AE1

Beyond reducing the need for as many wards tested in annual precision mapping for targeted planning, informants were mixed on whether they thought targeted planning using this model would eventually increase or reduce the overall costs of MDA intervention. The model outputs shown to informants (S3 Fig) predicted that the total number of treatments needed would slightly rise when using the geostatistical model [7]. An NTDCP staff member believed that in switching to targeted MDA, these costs would ultimately balance out:

“In terms of financial [resources], I don’t think if it will add much. Because… in terms of total budget…some communities…were over-treated and now they’re going to be dropped, then it will be balanced.”–NTDCP2

There were mixed opinions on whether the use of this model would create additional strain on financial resources currently available for planning. Some informants cited potential challenges to model integration that could arise in the need for more financial resources, specifically in the training of staff to use the model, as well as the cost of follow up studies to evaluate the model’s ability to make accurate predictions:

“At the end of the day, if we want to confirm what the model has told us, we need to dig in our pocket so…we can confirm [its accuracy].”–AE1

Additionally, informants believed that there would need to be sensitization when using this model, to justify, for example, why costs for drug distribution will have increased in an area that the model estimated would require treatment, despite it previously not believed to be endemic.

“The main barrier is going to be explaining [to] people why the treatment numbers may have gone up, and why the cost may have gone up in turn…That’ll be the main thing, because in situations like that people may ask, ‘what do you mean, you’re going down to the ward level, but we have to treat more.’ That’s going to be a very difficult concept to communicate.”–AE2

Finally, an informant working as an implementing partner believed that the maps generated by the model (shown in S2 and S3 Figs) could provide incentive for private partners working on MDA in Tanzania to use the model as well. They asserted that the maps could be used to generate more funding for schistosomiasis MDA and other services in some districts by demonstrating prevalence estimates in locations that have not previously had prevalence testing:

“Funding from the district [and] from…other government sources are there, but you need to prove…beyond reasonable doubt that the problem [of disease prevalence in a given area] exists. … Seeing it through data…just presented as one which is going to … [help to] secure funding from the government sources.”–IP2

### Services and operation capacity (Human resources, medicines, drugs, surveillance systems)

Key informants were asked questions about how they perceived the operational capacity of programs to use the model within existing services. Recurring themes within the interviews included human resources, precision mapping and calculating the number of PC drugs needed.

Key informants did not remark that use of a predictive model for decision-making would require a substantial amount of additional human resources, but a few commented on the importance of having modeling expertise within Tanzania to facilitate a sustainable process where modeling is truly integrated into the program:

“We need to have people within the program with such capacity to make sure that any time… we need this…kind of information we can be able to [model] it ourselves. So, training and capacitating people in the program is critically important.”–IP2

Here, the implementing partner highlights the importance of investing in training and capacity building of local staff for routine modeling to be sustainably integrated into programming. In addition to training local NTD workers on use of this model, almost all key informants regarded sensitization as a key consideration to integrating the use of a prediction model into services at different levels of schistosomiasis control. The informants had disparate views, however, on whether use of the model would facilitate or create challenges for sensitizing communities about the decision to provide disease control interventions in their areas. An implementing partners commented on the challenge of sensitizing communities that will not be selected to receive PC treatment:

“I’m sure the other ones that do not get…the intervention [will] be wondering why they didn’t. We get that even with…community MDA, [community members] kept asking…why didn’t you go to ours?…Some of them understand, but others don’t.”–IP1

This challenge has been specifically encountered by implementing partners with the delivery of targeted community MDA, which has happened in some areas on a limited basis. Informants posited that using models to select certain communities and not others could further challenge that communication. Another MDA implementing partner elaborated further on how this problem could be accentuated in targeted planning when communities have some cases of the disease but are not believed to be above the 10% prevalence threshold to need treatment:

“There could be some risk, because with the model…you leave [wards] without interventions…And because [a] ward…will be left [untreated], it doesn’t mean that there is no disease at all. There is disease there, …though, at [a] small scale…that could raise questions among the communities: ‘Why we are treating this ward [if] you are not treating [that] ward’. So, we need to get ready, …explaining details, the reasons and the factors behind choosing one ward and leaving the other.”–IP2

One of the implementing partners, however, believed that having model estimates at a ward level, in the absence of ward-level prevalence data, could actually help with sensitization of community leaders and MDA organizers in these scenarios:

“The information that will…simplify the advocacy and sensitization wherever you go to the community and for sensitization before you conduct MDA. So, because you will be having data… it [will] add more value and… increase impact and confidence as we plan for MDA in those areas that the model is showing they need interventions…so when…we show the visual picture…and when we [demonstrate to] them in a language that they understand…it becomes easier for them to accept the intervention.”–IP2

Informants furthermore asserted that another area where programs could be incentivized to use the model for ward-level estimates was for the calculation of the total number of treatments required. Each year, the national program must make this estimate and acquire and mobilize millions of treatments. With WHO guidelines recommending bi-annual treatment in high-risk communities, in addition to targeted treatment, there is concern that this calculation can become even more cumbersome. A key informant from the WHO predicted that using such model outputs could be beneficial for supporting country teams to make these calculations, and in turn make timely requests from pharmaceutical companies:

“We have seen this situation this year with the medicines request submitted by countries…and they are still submitting the medicines request at [district] level, but applying the new guidelines so that we have doubled or even sometimes tripled the request of praziquantel. And obviously that’s something that cannot be [sustained], as the pharma is not willing or keen to support. So, we need to obviously bring the countries to new schemes where we can improve the granularity when it comes to identifying areas endemic for schisto[somiasis], and I think this is where the endemic mapping becomes critical.”–WHO

One more operational consideration in using such models, brought up by WHO and one of the academic expert informants, was that that use of credible or confidence intervals surrounding model predictions could support teams in deciding where to hold interventions. When presented to informants, the geostatistical model outputs predict a single value estimating the mean prevalence in a ward, which the informants cited, could be challenging for decision-makers when the disease burden was predicted near cutoff ranges for treatment. The informants believed that with intervals surrounding estimates, it would better operationally facilitate decision-makers in justifying the selection of model informed locations for MDA with a higher level of confidence.

### Policy, planning and coordination

Five of the seven key informants were working within Tanzania at the time of the interview, either as part of, or in collaboration with, the NTDCP. Each of these informants were consistent in describing a system where the location of MDA campaigns and precision mapping surveys were largely selected at the central level, then subsequently passed down to district staff to implement. In addition to using prevalence data to guide decision-making, central level planners take stock of the different donors and implementing partners available throughout the country before creating plans and communicating them back down to the district level.

A NTDCP staff member and an academic expert, both of whom had been involved in central level coordination, did not feel that there would be any major barriers in integration of using prediction models to support this planning process in the future, but that if it was going to be routinely used and sustainably integrated, it would have to be integrated into national strategic guidelines. At the time of these interviews, Tanzania did not have national strategic guidelines, but instead used schistosomiasis control guidelines set forth by the WHO. The lack of a national strategy document or strategic guideline specific to Tanzania, is then a key barrier in integrating these models, or any other new intervention not currently in WHO recommendations.

“We don’t have the strategic document for the program in the country…basically we are missing the guide for the country. Where are we heading? Which strategies are we implementing? They are not documented. So … the goals for the national program [are] not clear…For me that is actually [the] number one challenge, and we need to have that written down. At least to show the road map where the country is going…we don’t have a very specific guideline for the country. Actually, we’ve been following the ones from the WHO.”–NTDCP1

In place of a national strategy document, the NTDCP instead adheres to guidelines from the WHO. The WHO guidelines and roadmap, however, do not specifically recommend use of prediction models in national programming, although they do call for more innovative strategies and new approaches to mapping disease burden throughout the country [13]. Informants thus felt the WHO more soundly advocating for the use of prediction models would ultimately lead to their integration and acceptance.

“If WHO can start using these models, that’s where you will really see the impact, they can have on country programs because their use will have been validated by an authority on this topic.”–AE2

“Incorporating the new guidelines from WHO, and also programmatically. What do we need? What do we need to achieve as a country? We need to have that. And we have already started discussing, because we are now starting drafting this [strategic document] for the schisto[somiasis] program. I believe a lot will come out of that document. And if this modeling would be one of the approaches, it would definitely be sustainable.”–NTDCP1

Implementing partners who work in partnership with NTDCP feel that once the NTDCP embrace using such models, it would be theoretically easy to put in place at lower levels.

“For this model to work better and get accepted, we need to have like an inception to these key decision-makers and policy makers at national and regional level. Once these […] people understand well the[se] kinds of models, it’ll be easy to trickle down and implement them at lower level where actually interventions are being done.”–IP2

In addressing why there are not currently explicit recommendations for use of predictive models in WHO guidelines, the WHO informant explained the challenges that come with recommending specific geospatial methods when there are many useful tools and models that might be beneficial in different contexts and settings. The informant asserted the importance of rigorously examining and harmonizing the best practices for each of these approaches integrating them into new guidelines.

“I think we need to try to standardize how we are going to use models to inform countries on schisto[somiasis] decisions. I think now there are different initiatives using different methodologies and I think it’s from a programmatic perspective, it is good to bring everyone together and try…to harmonize a framework… I think this is important because, at the end, these approaches are going to guide…decisions [at] country level. So, I think this is important that we discuss, for example, what type of models we think can better suit the program needs… what type of indicators […] are more useful for countries to make the decision.”–WHO

The WHO informant further acknowledged that countries can stand to benefit from using models to address the critical shortage of prevalence data, and that members of the WHO are aware that action is needed to put forth a set of recommendations for how these tools can better inform planning for schistosomiasis interventions.

Finally, several informants suggested that switching to more targeted planning for schistosomiasis would challenge the traditional practice of distributing drugs for both schistosomiasis and soil-transmitted helminths (STH) have been traditionally given together as part of a single MDA campaign. While this is an important consideration for disease control programs, which can more efficiently distribute drugs by coupling interventions for multiple endemic NTDs, this is less of a limitation of using a model-based strategy and more of an issue of using more targeted planning.

## Discussion

The primary objective of this study was to explore challenges and motivating factors associated with integrating a novel geostatistical model as part of the schistosomiasis control and planning process in Tanzania. KII conducted exposed a number of key challenges and motivating factors highlighted in Table 3, and weighed in on whether or not they believed that the introduction of the model could ultimately lead to a transition to more targeted planning for interventions.

**Table 3:** Overview of perceived incentives and challenges associated with integrating a novel geostatistical model to support planning for schistosomiasis control interventions in Tanzania.

Thematic Area	Perceived motivations for model integration	Perceived challenges with model integration
**Information Systems**	Strong agreement that a current lack of prevalence data inhibits the capacity to effectively plan targeted interventions like MDA	The model relies on existing data from information systems that may be outdated or inaccurate, which could lead to errors in prevalence predictions.
	Prevalence estimation can be very error-prone in the current system, as disease prevalence is often incorrectly assigned based upon neighboring district prevalence, or wards are assigned the prevalence of their corresponding district.	
	Informants believed that the model could improve the accuracy of identifying high-risk areas for targeted MDA, helping to allocate resources more effectively.	
**Financing**	Using this model could reduce the need for extensive, costly precision mapping surveys across all wards, lowering overall operational costs.	Concerns were raised about potential additional costs, such as training staff to use the model, follow-up studies to verify model predictions, and explaining increased drug distribution costs.
	Model-generated prevalence data could potentially be used to secure additional funding from government and other sources by demonstrating that certain districts are endemic	
**Services and Operational Capacity**	Informants generally believed that the maps generated by the geostatistical model can help guide targeted MDA and prevalence testing.	There may be variable preferences on how to make model outputs actionable and interpretable to planning staff. Some informants thought that the use of credible intervals may allow program staff to make better informed estimates with higher confidence on areas requiring treatment.
	Access to the model estimates can facilitate the estimation of total required treatments, which the NTDCP must request annually from donors and pharmaceutical companies	Implementing the model may require significant effort to educate and justify decisions to communities, especially when some areas are not selected for treatment despite having low-level disease presence.
	Mixed perceptions on whether the model would help with sensitization of communities when treating: suggestions that model outputs would provide an evidence base that would lead to greater acceptance	Investment in training and capacity building would be required for routine modeling and integration into existing programs.
**Policy and Planning**	The plans to create national strategy documents in the coming years could provide an opportunity for integrating novel tools like the geostatistical model.	There is currently no national strategy guideline. Therefore, the country program adheres strictly to WHO guidelines for planning. There are currently no recommendations or guidelines around using geostatistical models in the WHO handbooks. This makes them less likely to be used by country programs.
	Additional pressure from the WHO to switch to targeted interventions may incentivize country programs to adopt novel strategies like the geostatistical model	
**Coordination**		Transition to targeted planning for schistosomiasis may disrupt traditional NTD distribution strategies that provide drugs for multiple diseases.

The key informants unanimously agreed that limitations in information systems, specifically schistosomiasis prevalence data, was a key factor inhibiting a transition to more targeted planning. They also revealed that this lack of data has historically led to an error prone prevalence estimation method for which costly interventions like MDA are based upon. Informants believed that the geostatistical model could provide a more data driven solution to mapping prevalence and planning interventions. The importance of accurate disease prevalence mapping has been fundamental to disease epidemiology and control since the inception of the science, and in the context of NTDs. Robello and colleagues have asserted mapping is a “prerequisite for effective implementation of interventions against NTD” [29]. The description the systemic limitations in making data driven intervention site selections reinforces the problem statement that the model was built upon: that the paucity of prevalence data provides not only a programmatic incentive, but also need, to use alternative tools like the geostatistical model presented here to guide planning for communities in need.

While informants agreed with this notion, some cautioned that some of the information system elements feeding the model could also be prone to error. The geostatistical model includes elements like population density, which can change over time due to migration patterns and urban development across sub-Saharan Africa [30]. Additionally, several ecological components in the model, including the viability of schistosomiasis’ snail vector, can shift over time due to changing climate patterns [31]. However, even if the model predicted prevalence in an area where transmission is eventually no longer occurring due to cessation of water access, there may still be untreated populations in those areas from when transmission was occurring, and these people will need treatment with PC drugs regardless. Schistosomiasis prevalence patterns will likely shift over time in any scenario making it harder to locate cases, which aforementioned population shifts will likely only compound. While concerns related to such changes of model covariates are valid over a large enough timescale, they should not be a deterrent for using the model, but rather highlight the importance of updating the model routinely.

Financial implications are often one of the largest determinants of feasibility of integrating a new intervention. For chronically underfunded NTD programs, this is no exception. Informants spoke to financial concerns that could represent challenges to model integration, such as the costs that would be required to validate the model’s predictive accuracy. This is an important concern, as there should be an evidence base for the model’s performance to instill confidence and support in sensitization of the model to a wider audience. This can most effectively be done in a control trial demonstrating the model’s capacity to predict to a similar degree of accuracy as parasitological prevalence testing, but at a substantially lower cost. This may have better cross-sectoral appeal than presenting cross-validation metrics or mean squared error to assess the model’s performance. Past geostatistical modeling studies for schistosomiasis have demonstrated exactly that, showing that predictive accuracy can be nearly as good as parasitological testing and that by building a lattice with a mixture of conventional school based parasitological prevalence testing mixed with geostatistical model predictions, comprehensive estimates can be generated at a substantially lower cost [14].

If Tanzania is to have comprehensive prevalence estimates that would allow for prioritization of targeting interventions to the highest transmission risk areas in the future, using precision mapping alone is not a financially feasible option. To make this possible with the resources available, informants acknowledged that model estimates must be used, which should be an enormous motivating factor for Tanzania and other country programs to integrate such models into their planning process. In the model’s absence, programs will continue resorting to assigning neighboring area prevalence estimates, and using ecological zones to predict high prevalence, as explained by key informants. Further demonstration of cost savings of conducting targeted planning in this way would be an essential next step to incentivize the national program to integrate the model in routine planning. Finally, informants further believed that supplying prevalence estimates at a granular scale and providing the maps of areas estimated to require treatments in S2 and S3 Figs could enable municipalities and communities affected by schistosomiasis to request funds and stores of PC drugs available at district level. While this may not further incentivize the national team to use the model, it could be a large perk for district and sub-district health staff to claim interventions at the level needed, and support local action against schistosomiasis, sharing a common cause with the national team to drive down transmission in affected areas.

Informants generally believe that the geostatistical model estimates could offer programmatic incentives by supporting ongoing control activities, including: the selection of locations for MDA, selection of sites for prevalence testing, as well as for the annual calculation of medications required for treatment (S3 Fig). The provision of annual calculations facilitated by using model estimates and population registers to more quickly estimate treatment needs is a big advantage that could incentive program staff to integrate the model, as informants explained that national programs have historically been challenged by needing to calculate this yearly. Innovations that streamline workflows and make tasks easier for health professionals have been demonstrated to be more likely to be adopted and integrated into existing public health frameworks [32].

When discussing the best way to operationally use model estimates to guide intervention site selection, some informants believed that providing mean estimates of coverage, as was presented to key informants in the demonstration of model outputs mapped in S2–S3 Figs, would be more challenging for program staff than if credible intervals were provided. The rationale behind supplying credible intervals, per informants, was that it would then enable program staff to make site selection with a higher level of confidence if they, for example, filtered out all sites for treatment where the lower boundary of the interval did not exceed the 10% treatment threshold. The importance of supplying confidence or credible intervals is additionally recommended by NTD modelling consortium, who advocate for communication of uncertainty as one of their five principles [33]. While this will be important to do in the future, we still advocate that when using maps to represent areas where treatment is indicated, it is easier to do using a single value, in the case of this demonstration when represented by the mean.

Another key operational element that could challenge the integration of the model is the lack of local capacity to use and construct such models. Key informants explained that capacity building is not only needed to interpret and use model outputs, but to recreate the model in future iterations to circumvent the issues of the model using outdated population or climate data. Until there is a policy and incentive to use these models however, programs have little incentive to direct limited resources into creating such capacities. While major donors have begun to commit streams of capital toward strengthening local capacity in modeling, what will motivate overstretched and busy programs that do not have time to commit individuals and resources to electively use models will likely be a policy shift where these changes are strongly recommended as part of routine programming. Such a transition will likely pose operational challenges initially, while ultimately allowing for the development of experience and eventually capacity in autonomously using such models to supplement activity.

Informants recognized both potential challenges and opportunities when it comes to sensitizing the model to various actors involved in the planning and administration of treatment. Implementing partners felt that being able to demonstrate maps of ward-level model estimates could improve sensitization when programs need to communicate to community leaders why a ward is or isn’t receiving annual treatment. Community trust is integral to program sustainability, as previously research has found mistrust to be a factor associated with sub-optimal MDA coverage [34–36].

The key challenges affecting uptake and use of this model, flagged by informants, were in policy and planning, specifically that use of models were not currently integrated into any kind of national or international planning or strategy document, guideline or set of recommendations. As described in the results, the absence of a national guideline leads national planners to adhere to WHO guidelines on schistosomiasis control [37]. As national, academic, and WHO experts agreed, if these models were advocated for in such guidelines, their uptake in countries would be much more likely. In the absence of explicit recommendations on use of models in both the guideline and roadmap documents [13,37], however, they are less likely to be used by country programs. This creates a bottleneck in moving forward with innovative and novel strategies to treating schistosomiasis and other NTDs in Tanzania: national NTD programs wait for the WHO to provide recommendations on methods of mapping prevalence and targeting MDA, while the WHO advocates for country teams to pilot new approaches and methods to support with this problem. Countries are pressured to move to a system of targeted treatment with insufficient funding to conduct comprehensive parasitological surveys, and no recommendations on other methods of estimating prevalence in its place.

Until there are major health system improvements in surveillance systems and sentinel sites for reporting schistosomiasis prevalence, there are conceivably a limited number of options for obtaining ward-level data necessary to enable a data-driven operational approach to targeted interventions. The first option would be to conduct comprehensive parasitological surveys across the wards in the country, which informants agree is not financially or operationally feasible. The second would be to integrate and use some kind of estimation tools, like the models examined in this study, to supplement existing parasitological surveys to supply estimates of disease prevalence of wards.

Importantly, the transferability of our findings extends beyond the Lake Zone of Tanzania. Settings with limited prevalence data and reliance on WHO guidelines for MDA planning and decision-making can benefit from the integration of geostatistical modeling outputs. The relevance of our study is not limited to schistosomiasis control alone, but it also contributes to the examination of sustainability within NTD programming, as the integration of various novel interventions will be crucial for long-term success.

### Recommendations

Results from this study, which highlight the informants perceived value of using this model, along with their perceptions on national schistosomiasis program’s reliance on WHO guidelines to shape control strategies, should provide additional incentive for the WHO to begin developing methodological guidance on how such models can best be used by country programs. As countries look to the WHO, and other associated multilateral agencies such as the Expanded Special Project for Elimination of NTDs (ESPEN) for leadership on this matter, it is essential that alternative recommendations for estimating prevalence to supplement extremely costly parasitological surveys are provided. As geostatistical methods are increasingly being investigated to support planning of schistosomiasis control [7,14] as well as for a variety of applications related to planning and resource allocation for other PC preventable NTDs [21–23,38], their application to support mapping should be thoroughly investigated and integrated into recommendations where evidence indicates necessary. The absence of such recommendations may inhibit country progress on moving to targeted MDA and more effectively using limited schistosomiasis control resources in areas that need them most. Furthermore, the lack of such recommendations, and national policies that stem from them, will likely continue to stagnate capacity building in NTD modelling, since overstretched programs do not have resources to electively transition to using models. When model outputs are presented to programmatic staff, they should be clear, actionable, and include a measure of uncertainty like confidence or credible intervals. Models should be updated every few years to ensure that they are using the most accurate population and climate data available.

It is key to outline that this paper does not advocate for these methods to replace, but rather to supplement parasitological surveys, to reduce cost and allow for a more sustainable method of routinely evaluating prevalence at a sub-district level. We furthermore recommend studies to evaluate the performance of geostatistical and other models, so that their strengths, limitations and predictive accuracy can be better understood.

Finally, there is a paucity of research surrounding the sustainable integration of novel tools and strategies into NTD programming. We recommend further research that investigates the costs and benefits of using models and targeted planning, along with investigation into how novel interventions can be sustainably integrated into national NTD programs.

### Limitations

One of the limitations of this study was the relatively small sample size. While ideally a study like this would have included more informants, there is a small number of individuals involved in planning and decision making for schistosomiasis MDA in Tanzania. The informants who participated in this study included most of the key personnel involved in the MDA decision-making in Tanzania. It would be advantageous in subsequent studies to evaluate perceptions of key personnel involved in MDA decision making in several country programs, as this could effectively identify themes across endemic regions that can provide more generalizable insights into challenges and motivating factors of using geostatistical models. Additionally, the performance of the USAID NTD sustainability framework employed in this study had certain limitations. Despite its effectiveness in identifying the main relevant themes, there were instances where inductive coding was needed to address thematic gaps.

## Conclusion

Key informants involved in schistosomiasis control and MDA planning in Tanzania believe that geostatistical modeling outputs can help bridge data gaps and enable schistosomiasis control programs to conduct targeted MDA. While there are considerable financial and operational incentives for tool integration, challenges related to policy, planning, and the absence of specific guidelines need to be addressed. With detailed WHO guidance on the use of such models alongside precision mapping, geostatistical models can be added to an arsenal of decision-making tools that facilitate a better understanding of the geographical distribution of schistosomiasis prevalence in Tanzania, ultimately allowing for a more targeted and effective use of resources.

## Supporting information

S1 Text Overview of the questionnaire and interview structure. These questions demonstrate some of the baseline questions posed to participants, with some substitution and addition of questions depending on the role of the participant. With all participants, the interviews followed the same structure of asking questions about role and perceptions of MDA prior to demonstrating the model and its outputs.
(DOCX)

S1 Fig Presentation of model components as seen by key informants. The different covariates associations with cases of schistosomiasis, and methods of designing the geostatistical model were demonstrated to participants.
(TIFF)

S2 Fig A demonstration of the model outputs, and how their treatment areas differed from that in a conventional district level approach. A key component of this figure was demonstrating how the model provided comprehensive prevalence estimates of wards within the lake zone. This was not the exact figure shown in interviews, however, as a base layers from the previous map could not be published in this journal due to copyright issues. This figure was created in QGIS 3.30.1 using open-source spatial data from GADM.org, and is presented on open-source base layers from the United States Geological Survey (USGS). This figure was previously published by Mathewson et al. in a 2024 PLOS NTD manuscript [7].
(TIFF)

S3 Fig Implications for number of treatments required in conventional treatment vs using the geostatistical model outputs, as seen by key informants during interviews. This demonstration was intended to help the informants understand that using the geostatistical model would not necessarily create an indication for fewer treatments, and further demonstrated that model-based treatment recommendation areas would differ widely from the conventional district level approach. The maps were created in QGIS 3.30.1 using open-source shapefiles and spatial data from GADM.org. Data were presented on open-source base layers from the United States Geological Survey (USGS).
(TIFF)
