# Supplementary material for: Challenges and motivating factors for integrating geostatistical models in targeted schistosomiasis control: A qualitative case study in Northwestern Tanzania
Source: PLoS Negl Trop Dis. 2024 Dec 30;18(12):e0012770. doi: 10.1371/journal.pntd.0012770 (PMC11723622; doi:10.1371/journal.pntd.0012770)
Supplement: S1 Text — These questions demonstrate some of the baseline questions posed to participants, with some substitution and addition of questions depending on the role of the participant. With all participants, the interviews followed the same structure of asking questions about role and perceptions of MDA prior to demonstrating the model and its outputs. (DOCX) [file pntd.0012770.s001.docx]

*Through this interview we hope to gain better understanding of the enablers and barriers for integrating model-based tailoring of MDA at ward-level instead of district-level. The structure of the interview is as follows. We’ll first briefly discuss the current policy and practices and your role regarding policy and practice of MDA against SCH globally and in Tanzania if applicable. Then the PowerPoint slides shared with you in advance will be presented, after which we will discuss the prediction model and the integration of model-based tailoring of MDA.*

**Part I: Current policy and practice (max. 10 min.)**

1. Can you first describe your role, and work that you have done relating to MDA and SCH prevention and treatment?
2. How would you (briefly) describe the current practice of planning and execution of MDA against SCH?
3. From your perspective working on SCH control, what have been the main challenges in organizing MDA for schistosomiasis?

**Presentation** **of model insights and implications (max. 15 min.)**

1. **Overall benefits:** From your perspective, what would challenges or incentivize programs to integrate the geostatistical model for MDA?

**Part II: Integration of tailored MDA at ward-level into the SCH control system (max. 45 min.)**

Evaluation and adaptation

1. **Monitoring:** Currently, district-level surveillance and planning of MDA is done through precision mapping. How would you assess the reliability of currently used precision mapping data to make strategic decisions about MDA planning at district-level?
2. Overall, do you think the currently applied SCH monitoring and evaluation procedures hamper integration of ward-level model-based MDA using the geostatistical model, and if yes, how?

Policy and leadership

1. **Policy:** broadly spoken, would integration of the geostatistical model to guide MDA require policy change at national and sub-national level, and currently spelled out roles and responsibilities? If yes, in what way?

Delivery systems

1. **Management:** How would you assess the available systems and processes established for the government to manage all aspects of integration of the geostatistical model to guide MDA?
2. **Planning:** What MDA planning procedures characterize the SCH control system that might hamper integration?
3. **Integration:** *In previous MDA campaigns, activities were conducted in coordination with MDA activities for other NTDs, for example STH. This included planning exercises, community sensitization and media campaigns, co-distribution of drugs and vaccines, and monitoring and evaluation.*

To what extent does this count for activities for SCH MDA, and what would that mean for the integration of the geostatistical model?

Organizational capacity

1. **Staff:** Would there be enough staff available, with necessary skills and expertise, to integrate the geostatistical model to steer targeted MDA?
2. **Facilities:** How would integration be hampered by the current allocation of resources, i.e. facilities and equipment?

Budget

1. **Budgets:** What finance procedures characterize the SCH control system that might hamper integration?

Policy and leadership

1. **Communication:** How is continuous policy communication from national, sub-national and community-level government organized, and in what way could communicating the change of policy, implied by the shifting towards integrated geostatistical model for targeted MDA, be a challenge?
2. **Leadership:** Do you believe that there are people in relevant National ministries who could generate interest for the adoption and implementation of a geostatistical model to target MDA and take on a leadership role, as so-called ‘champions’?

**Part III: Conclusion and suggestions (max. 10 min.)**

1. Overall, what do you think are the most important incentives and challenges for the integration of the prediction models?

S1 Text: Overview of the questionnaire and interview structure. These questions demonstrate some of the baseline questions posed to participants, with some substitution and addition of questions depending on the role of the participant. With all participants, the interviews followed the same structure of asking questions about role and perceptions of MDA prior to demonstrating the model and its outputs.
